# Dental age estimation using the London atlas– which tooth and which tooth stage predict age best (excluding 3rd molars)?

**DOI:** 10.1007/s00414-025-03557-4

**Published:** 2025-08-05

**Authors:** Wei-Xi Tan, Janet Ann Davies, Helen Mary Liversidge

**Affiliations:** 1 Dental Institute, Barts and the London School of Medicine and Dentistry, Queen Mary University of London, London, UK; 2 Present Address: Department of Paediatric Dentistry, Hospital Sultanah Aminah, Johor Bahru, Johor Malaysia

**Keywords:** Dental age estimation, Developing teeth, London Atlas, Radiography

## Abstract

**Objectives:**

Developing teeth are frequently used to assess the developing dentition and to predict age. The London Atlas of Tooth Development estimates age as a single value (midpoint of an age interval). The aim was to determine which tooth stage, tooth, or combination of maxillary/mandibular teeth best estimates age using the Atlas.

**Materials and methods:**

The sample was 946 archived panoramic radiographs (491 male, 455 female) of dental patients aged 3–16 years. Crown and root stages of 8323 developing permanent teeth on the left side were assessed. Dental age (DA) was calculated for each tooth using 3rd edition of London Atlas App. Mean difference (MD) and mean absolute difference (MAD) between estimated and chronological age (CA) for each age category and for each tooth was calculated. Student *t*-test was used to assess MD. A linear regression model assessed predictive strength of individual teeth and combination of teeth with collinearity expressed as variance inflation factor (VIF).

**Results:**

The second premolar, canine and central incisor showed high collinearity with other teeth; excluding them did not improve age prediction. Individual tooth stages of different teeth could estimate age best for ages 3 to 12.

**Discussion:**

No single tooth predicted age best for all individual age categories for this sample because a single tooth stage spans more than one year. Different teeth were accurate for different age categories but the second molars from both jaws showed small MD, MAD and lower VIF values than other teeth.

## Introduction

**Fig. 1:**
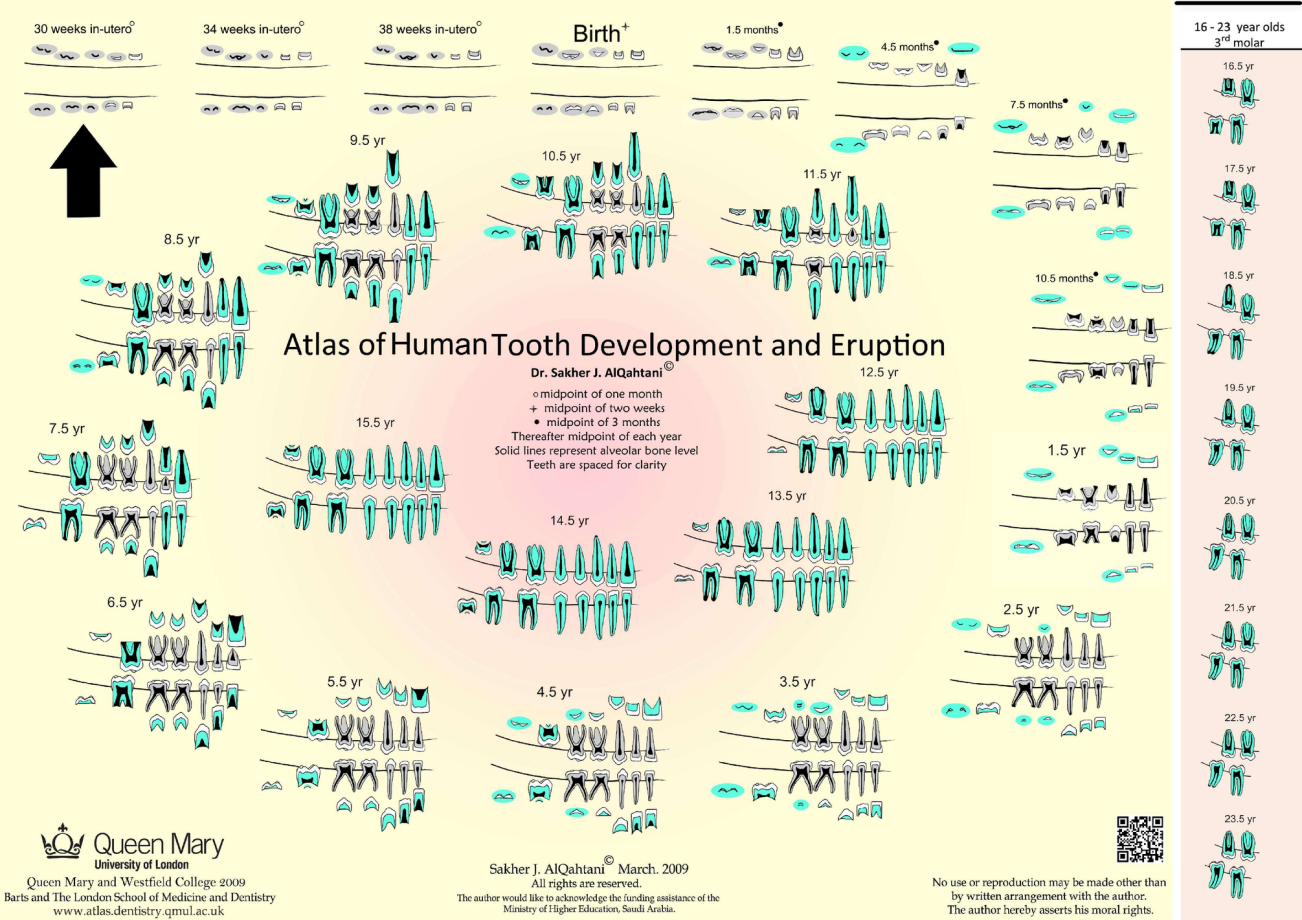
The London Atlas of Human Tooth Development and Eruption (Reproduced with permission from Sakher AlQahtani)

Age estimation in humans, either living or deceased, is required for matters related to births, adoptions, asylum seekers, and identification of age-at-death of the deceased in forensic cases [16, 19]. Chronological age (CA) is the number of days, weeks, months, or years an individual has been alive since day of birth. Biological age reflects maturation of a body system which is time-dependant and is irreversible (e.g., skeletal system, development of secondary sex characteristics, and the development of the dentition). Both chronological and biological age increase simultaneously but may differ due to variations in development. Dental age (DA), calculated from developing teeth can infer CA. Several approaches exist to obtain DA, one of which is the atlas method. The London Atlas [2] is a comprehensive, evidence-based atlas that is freely available in multiple languages and can be easily accessed via the website (https://www.qmul.ac.uk/dentistry/atlas/). It is tooth-specific and illustrates tooth development for 31 age categories. All age categories are illustrated, and tooth stages and eruption levels are described. Each drawing represents the median tooth and eruption stage for the age category (Eg. 3.5 years encompasses ages 3.00 to 3.99 years). Age categories are illustrated from 30 weeks in-utero to 23.5 years, and enamel, dentine and pulp are illustrated in different colours. The positions of teeth are illustrated with spaces in between which makes for clearer visibility (Fig. 1).

**Fig. 2:**
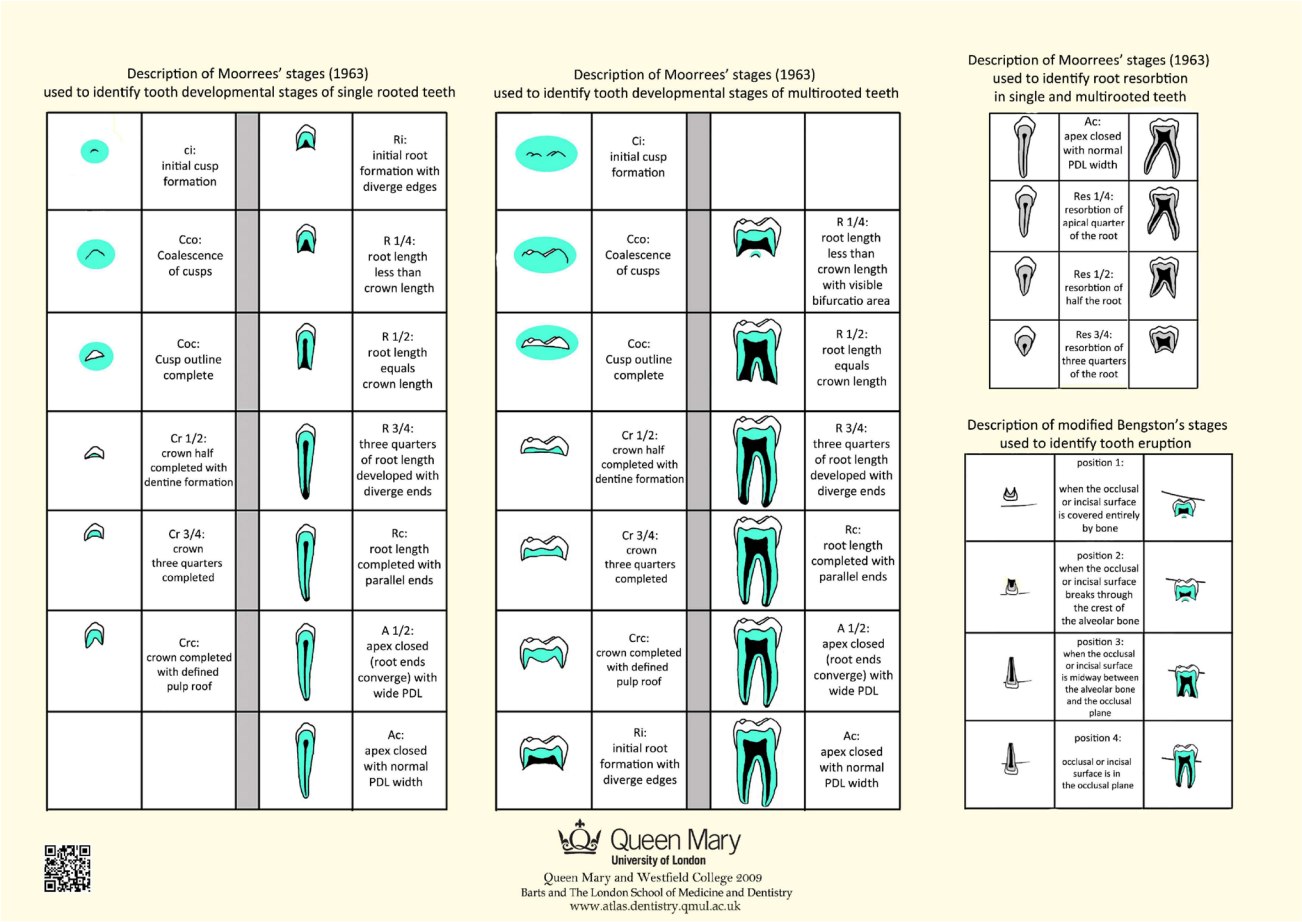
Moorrees’ stages of tooth development, and Bengston’s Stages of tooth eruption (Reproduced with permission from Sakher AlQahtani)

Tooth developmental stages according to Moorrees’ thirteen stages [14] for single-rooted and multi-rooted teeth are described in separate tables on the atlas to facilitate recognition of stages (Fig. 2).

It has been widely tested in world groups and results of a meta-analysis methods [9] show it to be accurate for most populations, with minimal discrepancies between the sexes and is easier to use than other dental age estimation methods [1, 3–8, 10, 15, 18, 20, 21]. The London Atlas is tooth specific, meaning that any developing tooth can be used to estimate age, however, it would be useful to know which tooth or combination of teeth is more accurate in assessing maturity or estimating age. The aim of this study was to assess which tooth, or combination of teeth (excluding third molars), was most accurate in predicting age using the London Atlas.

## Materials and methods

### Study design

This was a retrospective cross-sectional study testing the London Atlas app (3rd edition) to estimate age using dental panoramic tomograph (DPT) of individuals of known age. The radiographs had previously been taken for diagnostic and treatment purposes. Crown and root developmental stages of permanent teeth were assessed using the Moorrees’ stages of the London Atlas [14].

### Materials

The sample was 946 archived DPTs (493 male, 453 female) of dental patients aged 3–16 years of Bangladeshi and White British ethnicities who attended the Institute of Dentistry, Barts and the London School of Medicine and Dentistry, London (Table 1). These radiographs were first studied by Maber to test the accuracy of different dental age estimation methods [13]. Inclusion criteria were healthy individuals aged 3–16 years with good quality panoramic radiographs and immature teeth. Exclusions were individuals with hypodontia; supernumerary teeth; unusual pathology of the deciduous/permanent teeth or bone; history of chronic disease, systemic illnesses, syndromes and radiographs of poor image quality. Ethical approval was not required as archived anonymised radiographs were assessed.

**Table 1:** Age and sex of sample of archived dental radiographs (Maber sample)

Age in years	male	female	all
3.0–3.99	34	32	66
4.0–4.99	32	37	69
5.0–5.99	48	34	82
6.0–6.99	45	35	80
7.0–7.99	41	32	73
8.0–8.99	33	31	64
9.0–9.99	32	32	64
10.0–10.99	32	31	63
11.0–11.99	34	30	64
12.0–12.99	31	32	63
13.0–13.99	31	31	62
14.0–14.99	31	30	61
15.0–15.99	33	32	65
16.0–16.99	34	36	70
all	491	455	946

### Methods

The scanned radiographic images were viewed on a computer screen and tablet. Brightness, contrast, and magnification were adjusted to facilitate staging. All cases were numbered and assessed without knowledge of CA or sex. Each maxillary and mandibular tooth on the left side of each DPT was staged according to Moorrees et al.’s developmental stages (13 stages from initial cusp tips (Ci) to apex closure (Ac)) by the first author after training and calibration. Teeth on the right side were assessed if a tooth on the left appeared distorted or unclear.

Statistical analysis was carried out using IBM SPSS Statistics Data Editor Version 24. Weighted Cohen’s Kappa was used to test intra- and inter-examiner reliability. Intra-examiner reliability was calculated from re-assessment of 100 radiographs by the first author after a wash-out period of 3 months. Inter-examiner reliability was calculated from assessment of 20 radiographs, assessed by the first and second authors with a wash-out period of 1 week.

Crown and root stages of 8323 developing permanent maxillary/mandibular teeth on the left (or right) side were assessed. Immature teeth (prior to stage Ac) were included in the analysis. DA for each tooth was determined using the third version of the London Atlas App (using tables prior to availability of the software). All data analyses were for pooled sex. DA was subtracted from CA to give either a positive or negative result. Positive results indicate an overestimation whereas negative results indicate underestimation of age. Mean difference (MD) and mean absolute difference (MAD) between dental and chronological ages for each tooth, tooth stage as well as each age category were calculated. Student *t*-test was used to assess MD. A linear regression model was used to assess the predictive strength of individual teeth and combination of teeth. A high variance inflation factor (VIF) of > 10 was used to indicate multicollinearity.

Further results on the predictive strength of combination of teeth and results of eruption stages are available [17].

## Results

Intra-examiner reliability was 0.947 and inter-examiner reliability was 0.824 showing a strong level of agreement.

### Results by age category

Results of the mean difference between dental and chronological ages, analysed by age category are shown in Table 2. Only results of a non-significant difference of less than 0.5 year and where N was at least 10 are shown. Results showed that several teeth had more than one stage per age category. The age categories 5 to 7 years had the most individual tooth stages that could estimate age without bias and this number reduced with age. Age group 13 years and older had no results with mean difference less than 0.5 year.

**Table 2:** Tooth stages with small mean difference MD (< 0.5 years) between dental and chronological ages

Age Group	Tooth																											
	LM2				LM1				LP2				LP1				LC				LI2				LI1			
	Stage	N	MD	SD	Stage	N	MD	SD	Stage	N	MD	SD	Stage	N	MD	SD	Stage	N	MD	SD	Stage	N	MD	SD	Stage	N	MD	SD
3	Cco	17	−0.22	0.17	Crc Ri R1/4	29 13 23	0.03 −0.03 −0.11	0.25 0.31 0.32					Coc	18	0.07	0.23					Cr3/4	22	0.00	0.23	Crc	40	−0.05	0.24
4	Coc	23	−0.01	0.30					Coc	25	0.09	0.30					Cr3/4	36	0.05	0.31	Crc Ri	31 20	0.02 0.02	0.31 0.31				
5	Cr1/2	43	0.01	0.29					Cr1/2	41	0.01	0.29	Cr3/4	52	−0.01	0.30	Crc	47	−0.03	0.31	R1/4	37	−0.04	0.27	R1/4	52	−0.02	0.29
6	Cr3/4	38	−0.06	0.26					Cr3/4	32	0.04	0.28	Crc	32	0.01	0.27	Ri	14	0.10	0.25	R1/2	21	−0.14	0.26				
7	Crc	24	0.04	0.24					Crc	19	0.03	0.25	Ri	15	−0.01	0.23	R1/4	35	0.04	0.26	R3/4	31	−0.01	0.29	Rc	24	−0.04	0.28
8	Ri	20	0.01	0.29	Rc	35	0.03	0.28	Ri	24	0.10	0.31	R1/4	26	0.14	0.27					Rc	20	0.01	0.30				
9					A1/2	15	0.05	0.29	R1/4	19	0.10	0.26	R1/2	22	0.07	0.27	R1/2	14	0.00	0.30								
10	R1/2	24	−0.01	0.30					R1/2	20	0.12	0.31					R3/4	40	0.03	0.30								
11									R3/4	35	0.00	0.30	R3/4	32	0.04	0.31	Rc	13	0.06	0.32								
12	R3/4	37	0.05	0.25					Rc	14	−0.02	0.29																

	UM2				UM1				UP2				UP1				UC				UI2				UI1			
3					Crc	42	−0.01	0.27	Ci	21	0.02	0.25	Coc	18	0.03	0.23									C3/4	12	0.13	0.24
4	Coc	21	−0.01	0.30					Cco	13	0.23	0.27	C1/2	37	0.09	0.31	C3/4	34	0.10	0.32					Crc	31	0.13	0.32
5	Cr1/2	41	0.02	0.30	R1/4	38	0.03	0.30	C1/2	43	0.04	0.29	C3/4	48	−0.01	0.30	Crc	38	0.04	0.31	Crc	20	0.03	0.33				
6	Cr3/4	31	−0.05	0.31					C3/4	29	0.07	0.26	Crc	44	−0.01	0.25	Ri	35	−0.02	0.29	Ri R1/4	10 32	0.17 −0.03	0.30 0.27	R1/4	31	0.19	0.23
7	Crc	47	0.06	0.27					Crc	26	0.08	0.26	Ri	14	0.07	0.26	R1/4	28	0.04	0.27	R1/2	21	0.01	0.23	R1/2 R3/4	18 28	0.18 −0.04	0.25 0.27
8	Ri	18	−0.01	0.29					Ri	19	0.10	0.33					R1/2	23	0.04	0.33	R3/4	39	0.07	0.30	Rc	32	0.02	0.29
9	R1/4	32	−0.01	0.31					R1/4	22	0.03	0.30									Rc	18	−0.03	0.28				
10	R1/2	18	0.00	0.28					R1/2	18	0.08	0.30					R3/4	44	0.04	0.29								
11													R3/4	30	0.03	0.30												
12	R3/4	25	0.03	0.26					R3/4	22	0.07	0.23																

**Fig. 3:**
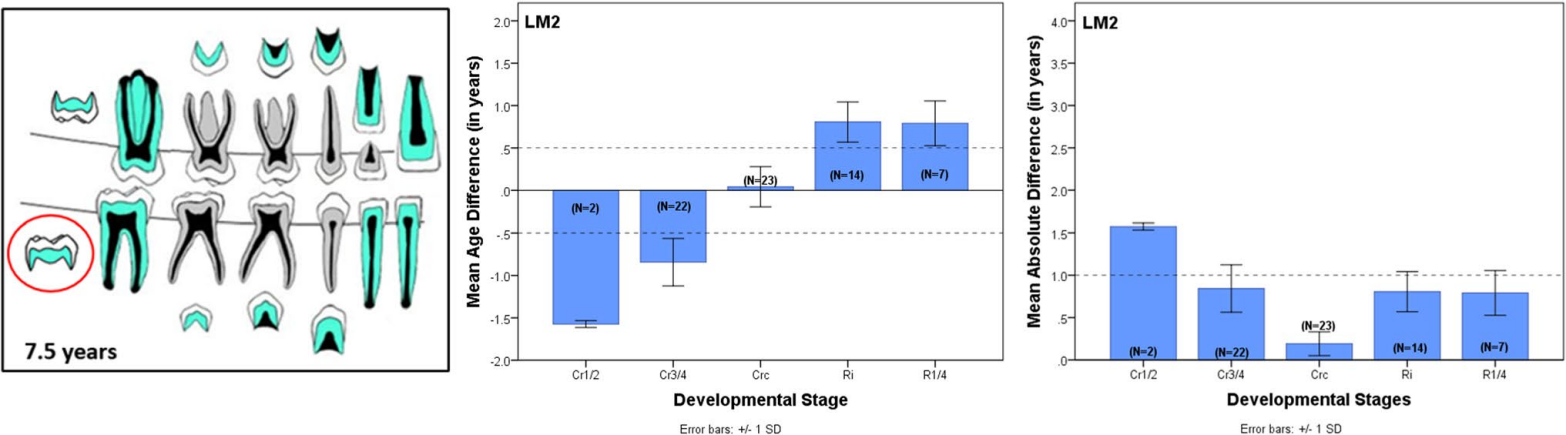
Example of analysis of LM2 (circled) at age 7.5 years of the Atlas (left). Middle bar chart shows results of mean difference between dental and chronological ages in years for seven-year-olds by LM2 stage. Stage Crc shows the least difference. Right bar chart shows the mean absolute difference by LM2 stage showing Crc has the smallest value

An example of the detailed results is shown in Fig. 3. This details the results for the mandibular second molar (LM2) in the 73 seven-year-olds of the Maber sample. On the left is the drawing for 7-year-olds from the Atlas with the LM2 circled. The middle bar-chart illustrates the average difference between dental and chronological ages by LM2 stage. The right bar-chart shows the MAD. LM2 stage Crc had the lowest average difference and smallest MAD and was the best stage to predict age for this age category.

**Fig. 4:**
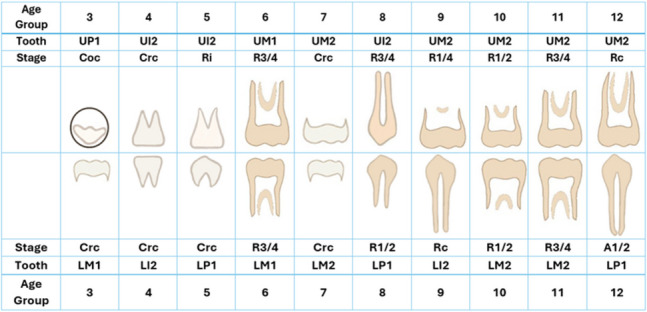
Drawing of individual maxillary and mandibular tooth stages that best predict age for specific age categories (based on smallest mean difference, least mean absolute difference and R squared value (see [17]))

Results of the MAD between estimated and chronological ages analysed by age category are shown in Table 3. Only results of less than 0.5 year and where N was at least 10 are shown. Age group 13 years and older had no results with MAD less than one year. Tables 3 and 4 show that several individual tooth stages of different teeth can accurately predict age for many age categories up to age twelve. The best tooth stage based on smallest MD, least MAD and highest R squared values (further details in [17]) are illustrated in Fig. 4.

**Table 3:** Tooth stages with small mean absolute difference MAD (< 1 year) between dental and chronological ages. Age group 13 years and older had no results with MAD < 1 year

Age Group	Tooth																				
	LM2			LM1			LP2			LP1			LC			LI2			LI1		
	Stage	*N*	MAD	Stage	*N*	MAD	Stage	*N*	MAD	Stage	*N*	MAD	Stage	*N*	MAD	Stage	*N*	MAD	Stage	*N*	MAD
3	Cco	17	0.24	Crc Ri R1/4	29 13 23	0.22 0.26 0.28				Coc	18	0.20				Cr3/4	22	0.19	Crc	40	0.21
4	Coc	23	0.25				Coc	25	0.27	Cr1/2	41	0.28	Cr3/4	36	0.28	Crc Ri	31 20	0.27 0.26			
5	Cr1/2	43	0.25				Cr1/2	41	0.25	Cr3/4	52	0.26	Crc	47	0.28	R1/4	37	0.23	R1/4	52	0.26
6	Cr3/4	38	0.23				Cr3/4	32	0.25	Crc	32	0.23	Ri	14	0.24	R1/2	21	0.24			
7	Crc	24	0.19				Crc	19	0.21	Ri	15	0.18	R1/4	35	0.22	R3/4	31	0.25	Rc	24	0.24
8	Ri	20	0.23	Rc	35	0.23	Ri	24	0.27	R1/4	26	0.25				Rc	20	0.25			
9	R1/4	38	0.59	A1/2	15	0.24	R1/4	19	0.23	R1/2	22	0.23	R1/2	14	0.24						
10	R1/2	24	0.26				R1/2	20	0.29				R3/4	40	0.26						
11							R3/4	35	0.26	R3/4	32	0.28	Rc	13	0.29						
12	R3/4	37	0.21				Rc	14	0.25												

	UM2			UM1			UP2			UP1			UC			UI2			UI1		
3	Cco	18	0.23	Crc	41	0.23	Ci	21	0.22	Coc	18	0.20							Cr3/4	12	0.23
4	Coc	21	0.24				Cco	13	0.30	Cr1/2	37	0.28	Cr3/4	34	0.30	Cr3/4	12	0.31	Crc	31	0.30
5	Cr1/2	41	0.26	R1/4	38	0.27	Cr1/2	43	0.25	Cr3/4	48	0.26	Crc	38	0.27	Crc	20	0.29			
6	Cr3/4	31	0.27	R1/2	22	0.27	Cr3/4	29	0.23	Crc	44	0.21	Ri	35	0.26	Ri R1/4	10 32	0.31 0.23	R1/4	31	0.28
7	Crc	47	0.24	R3/4	37	0.28	Crc	26	0.23	Ri	14	0.22	R1/4	28	0.23	R1/2	21	0.19	R1/2 R3/4	18 28	0.26 0.23
8	Ri	18	0.23	Rc	34	0.24	Ri	19	0.29	R1/4	25	0.28	R1/2	23	0.28	R3/4	39	0.26	Rc	32	0.24
9	R1/4	32	0.27				R1/4	22	0.26							Rc	18	0.23			
10	R1/2	18	0.24				R1/2	18	0.27				R3/4	44	0.26						
11										R3/4	30	0.26									
12	R3/4	25	0.21				R3/4	22	0.19	Rc	10	0.19									

Age group 3 indicates age 3.00 to 3.99 years etc. Other abbreviations see Table 2 legend

**Table 4:** Tooth stages with small mean difference MD (< 0.5 years) and small mean absolute difference MAD (< 1 year) in years between dental and chronological ages without age group split

	LM2			LM1			LP2			LP1			LC			LI2			LI1		
Stage	*N*	MD,SD	MAD	*N*	MD, SD	MAD	*N*	MD, SD	MAD	*N*	MD, SD	MAD	*N*	MD, SD	MAD	*N*	MD	MAD	*N*	MD,SD	MAD
Cco																					
Coc	37	0.03, 0.69	0.52							19	0.02, 0.29	0.23									
Cr1/2	95	0.03, 0.75	0.62				90	0.11, 0.74	0.61												
Cr3/4	96	0.04, 0.87	0.69				85	0.21, 1.06	0.80	107	–0.01, 0.77	0.62	98	0.12, 0.89	0.75	28	–0.18, 0.49	0.33			
Crc	63	–0.20, 0.98	0.76	31	–0.01, 0.29	0.25	48	0.18, 0.96	0.73	67	0.13, 0.78	0.60	121	–0.08, 0.90	0.75	84	0.14, 0.88	0.74			
Ri	54	0.07, 1.08	0.83	18	–0.25, 0.47	0.42	122			31	0.08, 0.71	0.54	36	0.05, 1.00	0.77				27	0.32, 0.97	0.81
R1/4													80	0.13, 0.93	0.71						
R1/2	51	–0.24,1.05	0.79																		
R3/4																					
Rc				103	–0.07, 1.21	0.93										54	–0.18, 1.07	0.83	51	–0.17, 0.84	0.65
A1/2				42	–0.02, 1.22	0.92															

	UM2			UM1			UP2			UP1			UC			UI2			UI1		
Ci							25	–0.14, 0.45	0.33												
Cco																					
Coc	41	–0.02, 0.72	0.56							20	–0.03, 0.30	0.24									
Cr1/2	86	0.12, 0.69	0.58				87	0.05, 0.70	0.57												
Cr3/4										95	0.09, 0.70	0.58							17	–0.25, 0.72	0.50
Crc	121	0.08, 1.08	0.84				68	0.18, 1.00	0.78	90	0.01, 0.79	0.60									
Ri	56	–0.24, 1.10	0.87							28	0.18, 0.88	0.64									
R1/4																88	0.17, 0.94	0.75			
R1/2													65	0.18, 1.10	0.89	41	0.21, 0.82	0.63			
R3/4																97	0.20, 0.99	0.80	67	–0.02, 0.93	0.72
Rc				112	–0.10, 1.20	0.97										45	0.05, 1.05	0.80	77	–0.16, 0.97	0.77
A1/2				10	0.33, 0.71	0.67										20	0.42, 1.21	0.98			

Only NS results reported where mean difference is < 0.5 year, MAD < 1 year and *N* at least 10. Abbreviations see Table 2 legend

### Results by tooth stage

Results of the MD and MAD between dental and chronological ages by tooth stage (disregarding age categories) are shown in Table 4. Only results of a non-significant difference of less than 0.5 year, MAD of less than 1 year and where N was at least 10 are shown. LM2 was the tooth with most stages with small MD and small MAD followed by the maxillary lateral incisor (UI2) root stage.

### Results by tooth

Results of the MD, MAD between dental and chronological ages by tooth (all stages combined) are shown in Table 5. MD values of mandibular teeth were smaller than maxillary teeth although values for the second molar were similar between the jaws. MAD values were similar between the jaws. The mandibular first premolar (LP1) and both maxillary premolars MD values were the highest of all teeth. VIF values for all premolars were high showing collinearity. The first molars (LM1, UM1) and lower central incisor (LI1) had smallest VIF values.

**Table 5:** Mean difference, mean absolute difference between dental and chronological ages and variance inflation factor for individual teeth

Tooth	N	MD, SD	MAD	VIF	Tooth	N	MD, SD	MAD	VIF
LM2	765	−0.01, 1.09	0.85	6.050	UM2	734	0.07, 1.14	0.87	5.663
LM1	496	0.07, 1.04	0.81	4.118	UM1	464	0.32, 0.97	0.81	4.688
LP2	693	0.17, 1.11	0.88	10.146	UP2	624	0.51, 1.11	0.93	8.673
LP1	666	0.35, 0.97	0.81	10.224	UP1	630	0.50, 0.98	0.86	11.083
LC	655	0.12, 0.93	0.93	5.781	UC	655	0.24, 1.07	0.89	7.855
LI2	450	−0.20, 0.91	0.73	5.866	UI2	498	0.42, 0.98	0.87	5.256
LI1	395	0.08, 0.93	0.72	4.527	UI1	466	0.37, 0.93	0.82	5.740

*N* number of developing teeth, *MD* mean difference in years, *SD* standard deviation, *MAD* mean absolute difference in years, *VIF* variance inflation factor

### Combination of teeth

**Fig. 5:**
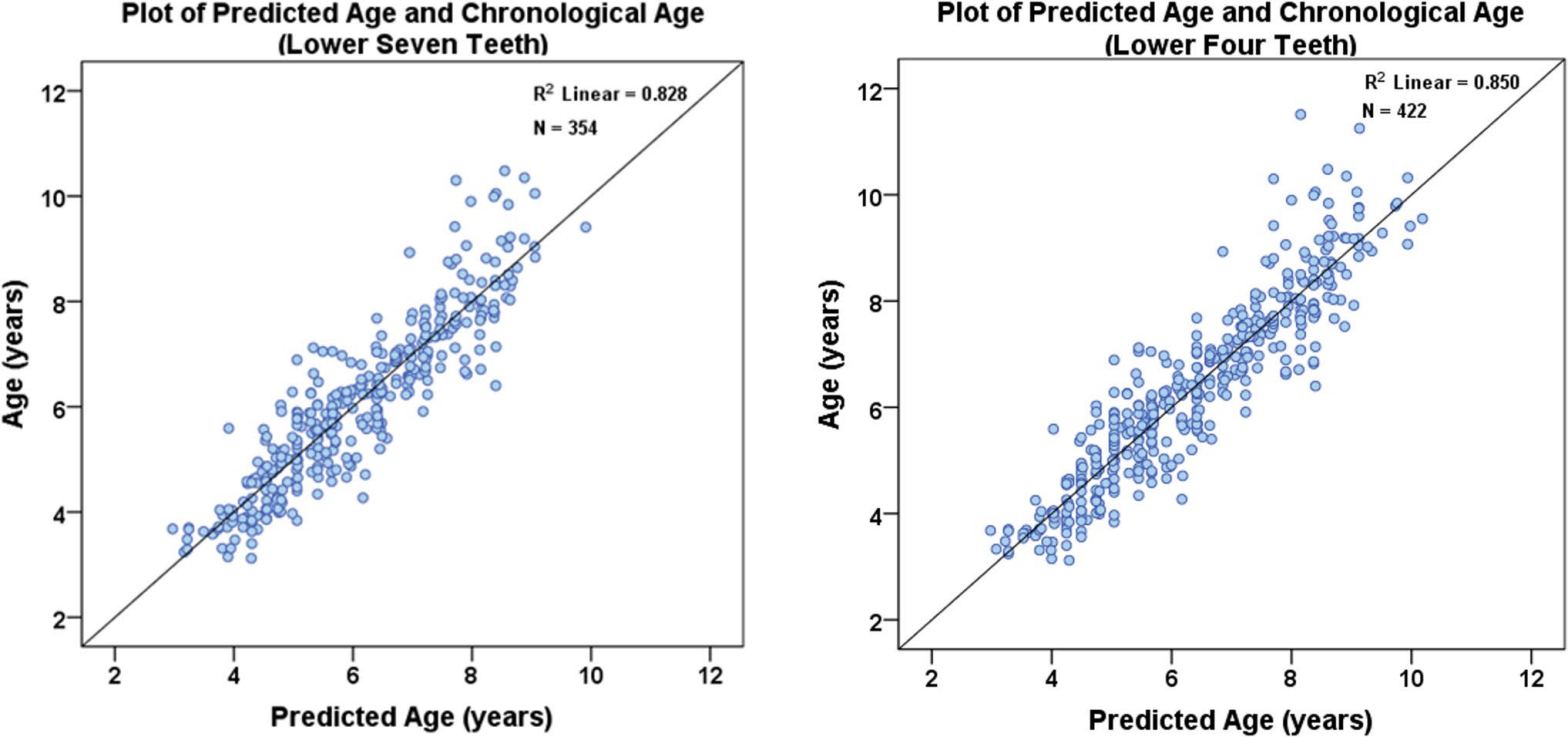
Plot of predicted values from multiple regression of seven versus four mandibular teeth and age. Left all seven teeth, right four teeth (LM2, LM1, LP1, LI2)

As multiple teeth estimated age with low bias and low inaccuracy for each age category, we aimed to find out which tooth was the best predictor for each age category. To ascertain multicollinearity between teeth, forward regression was used on maxillary and mandibular left teeth with CA as the dependent variable, and DA as independent variables. Teeth with p < 0.05 are statistically significant contributors to the model. Based on the regression models, all premolars from both arches had high VIF values (> 10), indicating collinearity. In addition, second premolars (P2s), canines (Cs) and central incisors (I1s) of both arches had p-values > 0.05 and excluding them did not change the age predictive value. This meant that, using only “M2, M1, P1 and I2” from either arch was as accurate as using all seven teeth. This is illustrated in Fig. 5.

In summary results from the *t*-test, two-way ANOVA and regression models found that both LM2 and UM2 showed low MD, MAD and VIF values indicating that M2s were the best individual teeth to predict age.

## Discussion

Estimating chronological age from dental radiographs is a core component of forensic and anthropological investigations. Most existing methods require assessment of multiple teeth within a single jaw or across both jaws. However, such approaches pose a problem when applied to incomplete dentitions, such as in archaeological remains or forensic cases where only one or a few teeth may be preserved. In these contexts, the ability to accurately estimate age from a single tooth becomes highly valuable. This study aimed to identify which tooth, or which combination of teeth, and at which stage of development is best at predicting age for 3 to 16-year-olds.

Our findings indicate that the predictive strength of teeth varies across age categories; different teeth were stronger predictors for different age categories. Specifically, no single tooth consistently emerged as the best predictor throughout the entire age range of 3 to 16 years. This variability may reflect the natural progression of dental development and the biological overlap (collinearity) between stages of adjacent or contralateral teeth. Notably, the second permanent molars demonstrated the highest coefficient of determination (R^2^) values with smallest confidence intervals suggesting their superior predictive performance for this sample of age range 3 to 16 years [17]. These findings align with previous research that emphasized the value of tooth-specific approaches, such as the work by Liversidge et al. [12], which also found increased accuracy when using maturity scores of individual teeth rather than mean of multiple teeth [12].

The performance of an age estimation method over a wide age range test sample is often expressed as a single value. A recent systematic review and meta-analysis of the accuracy of age estimation using the London Atlas found a standardised mean differences of 0.02 years for MD and 0.78 for MAD [9]. This is of interest,however, our method which evaluates specific tooth stages by age category, allows for more precise age estimation, particularly valuable when working with individual cases rather than population-level trends. The most accurate tooth stages per age category are useful when assessing dental maturity in a living child, but if age is unknown, then it is more useful to assign tooth stages of an individual and refer to which stages are most accurate in Table 4. *T *his enhances decision-making by linking morphological assessment directly to statistically grounded age estimates.

Our findings also contribute to the ongoing conversation about individual versus group accuracy. While group metrics provide general benchmarks, they are not directly transferable to individual assessments. In this study, several individual tooth stages—including the second molars and maxillary lateral incisor root development—showed low bias and small MAD values for age categories 3 to 12 years, supporting their diagnostic reliability. This may in part be due to the overlap between the age range of our test sample and the critical stages of second molar development.

However, our study is not without limitations. First, the Atlas method—in common with all methods based on age specific illustrations—estimates age as the midpoint of an age category rather than a precise point estimate. Another limitation is the choice of Moorrees’ tooth stages that include crown and root fractions. Tooth stages are discrete events in a continuum of the maturational process and information from longitudinal radiographs about the duration of individual tooth stages is largely undescribed. Some tooth stages are difficult to identify with certainty. Crown or root fractions must be subjectively estimated based on prior knowledge of normal crown height or root length. This can be challenging, particularly when a developing tooth is distorted or not entirely clear radiographically. Although experienced observers may mitigate this variability, it remains a source of potential error. Another limitation of our study is the pooling of males and females, although Seselj et al. [16] have shown that sex does not greatly influence age estimation. In addition, pooled-sex and opposite-sex reference data were almost identical to sex-specific reference data in age estimation performance [11]. Finally, the statistical limitation of evaluating all permutations of tooth combinations adds complexity to interpreting which combinations genuinely offer improved predictive accuracy, particularly in the presence of multicollinearity.

Despite these limitations, our findings underscore a key conclusion: no single tooth predicted age best across all age categories for our test sample. Different teeth were accurate for different age categories. The second permanent molar in both jaws demonstrate strong potential as reliable indicators for chronological age estimation during the mixed dentition period and should be prioritized in subsequent forensic research.

## Data Availability

The results that support the findings will be available on Queen Mary Research Online (https://qmro.qmul.ac.uk/xmlui/handle/123456789/108631).

## Permanent teeth

LM2: Mandibular second molar
LM1: Mandibular first molar
LP2: Mandibular second premolar
LP1: Mandibular first premolar
LC: Mandibular canine
LI2: Mandibular lateral incisor
LI1: Mandibular central incisor
UM2: Maxillary second molar
UM1: Maxillary first molar
UP2: Maxillary second premolar
UP1: Maxillary first premolar
UC: Maxillary canine
UI2: Maxillary lateral incisor
UI1: Maxillary central incisor

## Tooth stages

Ci: Cusp initiation
Cco: Coalescence of cusps
Coc: Cusp outline complete
Cr1/2: Crown half complete
Cr3/4: Crown three quarters complete
Crc: Crown complete
Ri: Root initiation
Cli: Cleft initiation
R1/4: Root length one quarter
R1/2: Root length one half
R3/4: Root length three quarters
Rc: Root length complete
A1/2: Apex half closed
Ac: Apex closed (mature root)
CA: Chronological age
DA: Dental age
DPT: Dental panoramic tomograph
MAD: Mean absolute difference between dental and chronological ages
MD: Mean difference between dental and chronological ages
SD: Standard deviation
VIF: Variance inflation factor
